# MicroRNA-1269 promotes cell proliferation via the AKT signaling pathway by targeting RASSF9 in human gastric cancer

**DOI:** 10.1186/s12935-019-1026-4

**Published:** 2019-11-21

**Authors:** Wen-Li Liu, Hu-xia Wang, Cheng-xin Shi, Fei-yu Shi, Ling-yu Zhao, Wei Zhao, Guang-hui Wang

**Affiliations:** 1 Department of Dermatology, The First Affiliated Hospital of Xi’an Jiaotong University, Xi’an, 710061 Shaanxi China; 2 Mammary Department, Shaanxi Provincial Tumor Hospital, Xi’an, 710061 Shaanxi China; 3 Department of General Surgery, The First Affiliated Hospital of Xi’an Jiaotong University, Xi’an, 710061 Shaanxi China; 4 Department of Cell Biology and Genetics, School of Basic Medical Sciences, Xi’an Jiaotong University Health Science Center, Xi’an, Shaanxi China

**Keywords:** miR-1269, RASSF9, Gastric cancer, Proliferation, Apoptosis

## Abstract

**Background:**

MicroRNAs (miRNAs) play key roles in tumorigenesis and progression of gastric cancer (GC). miR-1269 has been reported to be upregulated in several cancers and plays a crucial role in carcinogenesis and cancer progression. However, the biological function of miR-1269 in human GC and its mechanism remain unclear and need to be further elucidated.

**Methods:**

The expression of miR-1269 in GC tissues and cell lines was detected by quantitative real-time PCR (qRT-PCR). Target prediction programs (TargetScanHuman 7.2 and miRBase) and a dual-luciferase reporter assay were used to confirm that Ras-association domain family 9 (RASSF9) is a target gene of miR-1269. The expression of RASSF9 was measured by qRT-PCR and Western blotting in GC tissues. MTT and cell counting assays were used to explore the effect of miR-1269 on GC cell proliferation. The cell cycle and apoptosis were measured by flow cytometry. RASSF9 knockdown and overexpression were used to further verify the function of the target gene.

**Results:**

We found that miR-1269 expression was upregulated in human GC tissues and cell lines. The overexpression of miR-1269 promoted GC cell proliferation and cell cycle G1-S transition and suppressed apoptosis. The inhibition of miR-1269 inhibited cell growth and G1-S transition and induced apoptosis. miR-1269 expression was inversely correlated with RASSF9 expression in GC tissues. RASSF9 was verified to be a direct target of miR-1269 by using a luciferase reporter assay. The overexpression of miR-1269 decreased RASSF9 expression at both the mRNA and protein levels, and the inhibition of miR-1269 increased RASSF9 expression. Importantly, silencing RASSF9 resulted in the same biological effects in GC cells as those induced by overexpression of miR-1269. Overexpression of RASSF9 reversed the effects of miR-1269 overexpression on GC cells. Both miR-1269 overexpression and RASSF9 silencing activated the AKT signaling pathway, which modulated cell cycle regulators (Cyclin D1 and CDK2). In contrast, inhibition of miR-1269 and RASSF9 overexpression inhibited the AKT signaling pathway. Moreover, miR-1269 and RASSF9 also regulated the Bax/Bcl-2 signaling pathway.

**Conclusions:**

Our results demonstrate that miR-1269 promotes GC cell proliferation and cell cycle G1-S transition by activating the AKT signaling pathway and inhibiting cell apoptosis via regulation of the Bax/Bcl-2 signaling pathway by targeting RASSF9. Our findings indicate an oncogenic role of miR-1269 in GC pathogenesis and the potential use of miR-1269 in GC therapy.

## Background

Gastric cancer (GC) is considered to be one of the most prevalent lethal malignancies and the second leading cause of cancer-related death in the world, particularly in East Asia and South Africa [1, 2]. Most gastric cancers are diagnosed at advanced stages, when efficient therapeutic methods are limited [3]. The high recurrence and metastasis rate of GC is the biggest obstacle [4, 5]. Despite evident advances in the treatment of early GC, including radiotherapy, chemotherapy, surgical techniques, adjuvant therapy, molecular targeted therapy and earlier diagnosis, the 5-year survival rate of patients with advanced GC remains only 5–20% [6, 7]. GC pathogenesis is a multifactor, multistep, complicated process that is related to abnormal gene expression. However, the exact molecular mechanisms relevant to GC development and progression remain unclear. Hence, it is of great significance to further elucidate the potential pathogenesis of GC and look for new therapeutic targets for this disease.

MicroRNAs, also known as miRNAs, are endogenously expressed, small, single-stranded noncoding RNAs consisting of 19–25 nucleotides [8]. miRNAs may downregulate gene expression by binding to the 3′-untranslated regions (3′-UTRs) of specific target messenger RNAs (mRNAs), leading to inhibition of translation or mRNA degradation [9]. It has been reported that miRNAs participate in numerous important biological processes, such as cell survival, proliferation, cell cycle progression, differentiation, development, inflammation, metabolism, migration, invasion and apoptosis, as well as tumor development, metastasis, angiogenesis, and immune reactions [10–12]. miRNAs play an important role in regulating cancer-related gene expression in tumorigenesis. In GC, miR-144, miR-141, miR-338-3p, miR-361, miR-449a, and miR-638, among others were reported to inhibit the oncogenicity of tumors [13–15], and miR-19a, miR-425, and others were demonstrated to induce the oncogenicity of tumors [16]. Several studies have shown that miR-1269 is clinically significant and a potential biomarker that plays a crucial role in carcinogenesis and cancer progression in lung cancer and hepatocellular carcinoma [17–20]. Recently, we found that miR-1269 is one of the most frequently upregulated miRNAs in GC tissues and cell lines. However, the role of miR-1269 and its underlying mechanisms in GC remain unclear. Using bioinformatics software, we predicted that miR-1269 could target Ras-association domain family 9 (RASSF9). The RASSF family comprises 10 members from RASSF1 to RASSF10. One feature of this family is the Ras-association domain (RA), and this family can be subdi-vided into C-terminal (RASSF1-6) or N-terminal (RASSF7-10). It has been reported that the N-terminal RASSF genes are involved in cell growth, survival and apoptosis, among other processes [21]. Evidence suggests that RASSF9 inhibits breast cancer cell growth [22]. To date, the function of RASSF9 in many other cancers, including GC, has not been reported.

In this study, we investigated the function and mechanism of miR-1269 in human GC. We found that the expression of miR-1269 was dramatically upregulated in human GC tissues and cell lines. Furthermore, miR-1269 significantly promoted AGS and MKN-45 cell proliferation and cell cycle transition through the AKT signaling pathway by targeting RASSF9 via its 3′-UTR and suppressed AGS and MKN-45 cell apoptosis by activating Bcl-2/Bax signaling. Silencing RASSF9 had similar cellular and molecular effects as those caused by miR-1269 overexpression. These findings demonstrate an oncogenic role of miR-1269 in GC progression, suggesting that miR-1269 may be a novel therapeutic target for GC treatment.

## Materials and methods

### Human GC clinical samples

Seventy-three GC tissue samples and paired adjacent normal tissue samples were obtained from patients who were diagnosed at the Department of General Surgery, the First Affiliated Hospital, Xi’an Jiaotong University, China, between February 2016 and September 2017. We obtained informed consent from each patient before specimen collection. The collected tissues were immediately stored at − 80 °C. The experiments were approved by the Ethics Committee of The First Affiliated Hospital of Xi’an Jiaotong University.

### Cell culture

We purchased the human GC cell lines BGC-823, SGC-7901, MKN-45, and AGS, and the normal human gastric epithelial cell line GES-1 from the Cell Bank (Beijing, China). Cells were authenticated by the Cell Bank. The cells were cultured in RPMI-1640 medium (Gibco BRL, NY, USA) containing 10% fetal bovine serum (Gibco), penicillin (100 U/mL) and streptomycin (100 μg/mL) and were incubated at 37 °C in an incubator under 5% CO_2_.

### Quantitative real-time PCR (qRT-PCR)

Total RNA was extracted from human GC tissues and cultured cells with TRIzol reagent (Invitrogen, Carlsbad, CA, USA). PrimeScript RT Reagent Kits and SYBR Premix Ex Taq II Kit (Takara, China) were used to measure miR-1269 expression and RASSF9 mRNA expression. qRT-PCR was performed using the iCycler iQ Multicolor qRT-PCR System (Bio-Rad, CA, USA). The data were normalized to RNU6B (U6) or GAPDH gene expression. The primer sequences included the miR-1269 reverse-transcription primer (5′-GTCGTATCCAGTGCGTGTCGTGGAGTCGGCAATTGCACTGGATACGACTCAGGTC-3′), miR-1269 PCR forward primer (5′-ATCCAGTGCGTGTCGTG-3′), miR-1269 PCR reverse primer (5′-TGCTGGTCATCGTGCCGAG-3′), U6 reverse-transcription primer (5′-CGCTTCACGAATTTGCGTGTCAT-3′), U6 PCR forward primer (5′-GCTTCGGCAGCACATATACTAAAAT-3′), U6 PCR reverse primer (5′-CGCTTCACGAATTTGCGTGTCAT-3′), RASSF9 PCR forward primer (5′-ACAACAATCCCGCAGTTCAAA-3′), RASSF9 PCR reverse primer (5′-GTGTCTGGATTTCCAGGGTGA-3′), GAPDH forward, 5′-GCCACATCGCTCAGACAC-3′; GAPDH reverse, 5′-GCCCAATACGACCAAATCC-3′. The 2^−∆∆Ct^ method was employed in the qRT-PCR analysis.

### Expression vector construction and transfection

Hsa-miR-1269 precursor expression vector (named miR-1269) and the empty vector (named Control) were constructed using chemosynthetic oligonucleotides and were inserted into the pcDNA6.2-GW/EmGFPmiR plasmid according to the manufacturer’s instructions. Human RASSF9 gene DNA was inserted into the pCMV2-GV146 vector (GeneChem Co. Ltd, Shanghai, China). Transfection was performed using Lipofectamine 2000 (Invitrogen, Carlsbad, CA, USA).

### Anti-miR-1269/RASSF9 siRNA synthesis and transfection

Interfering RNA oligonucleotides served as miR-1269 inhibitors (named anti-miR-1269) and were synthesized by GenePharma (Shanghai, China). The sequence of anti-miR-1269 was 5′-CCAGTAGCACGGCTCAGTCCAG-3′. Scrambled siRNA served as the control (named anti-miR-Control), and the sequence was 5′-CAGUACUUUUGUGUAGUACAA-3′. The RNA oligonucleotides were transfected into GC AGS and MKN-45 cells with Lipofectamine 2000. Small interfering RNAs (siRNAs) were used to silence the RASSF9 gene. RASSF9 siRNA-1 (sense 5′-GAGAAUGAAAGAGCUGGAUTT-3′ and antisense 5′-AUCCAGCUCUUUCAUUCUCTT-3′), RASSF9 siRNA-2 (sense 5′-GAGGUUCUGAUAAGAAGUATT-3′ and antisense 5′-UACUUCUUAUCAGAACCUCTT-3′), and negative siRNA (NC-siRNA, sense 5′-AATTCTCCGAACGTGTCACGT-3′ and antisense 5′-ACGTGACACGTTCGGAGAATT-3′) were synthesized by GenePharma. The siRNAs were transfected by using Lipofectamine 2000 and diluted to 50 nM for the next experiment.

### Dual-luciferase assay

The binding site for miR-1269 in the 3′-UTR of RASSF9 was constructed with synthetic oligonucleotides (Beijing AuGCT DNA-SYN Biotechnology, China) and cloned into the pmirGLO Dual-Luciferase expression vector (named RASSF9-WT). The mutated 3′-UTR sequence of RASSF9 was also cloned and named RASSF9-MT. The pre-miR-1269 plasmids and reporter plasmids (WT or MT) were cotransfected into HEK293 cells. The cells were harvested and measured 24 h after transfection. The Dual-Luciferase Assay System (Promega, Madison, USA) was used to examine reporter activity according to the manufacturer’s protocol.

### MTT assay

Human GC AGS and MKN-45 cells (4000 cells/well in 100 μl RPMI-1640 medium) were plated in 96-well plates (5 wells/group) and incubated for 24 h. Next, the cells were transfected with Control, miR-1269, anti-miR-Control, anti-miR-1269, NC-siRNA (50 nM), RASSF9 siRNA (50 nM), vector control or RASSF9 expression vector for 24, 48 or 72 h, respectively. Cell viability was measured using an MTT assay FLUOstar OPTIMA microplate reader (Molecular Devices, Sunnyvale, CA, USA) at a wavelength of 492 nm.

### Cell counting assay

To detect cell proliferation, 2 × 10^5^ cells were plated in 60-mm-diameter plates and cultured for 24 h. AGS and MKN-45 cells were treated separately with Control, miR-1269, anti-miR-Control, anti-miR-1269, NC-siRNA (50 nM), RASSF9 siRNA (50 nM), vector control or RASSF9 expression vector. The number of cells was calculated at 24, 48 and 72 h after transfection with a Countess automated cell counter (Life Technologies Corp., Carlsbad, USA).

### Cell cycle analysis

At 24 h after transfection, AGS and MKN-45 cells were harvested by trypsinization for analysis. The cells were then washed twice with PBS and fixed with 70% ethanol at 4 °C. After washing again, the cells were dyed by using 50 μg/ml propidium iodide with 50 μg/ml RNase A for 12 min at room temperature. Finally, cell cycle distribution was measured through fluorescence-activated cell sorting (BD Biosciences, USA), and the proportions were analyzed with ModFit software (Bio-Rad Laboratories, Hercules, CA, USA).

### Apoptosis analysis

AGS and MKN-45 cells were plated into 6-well plates in triplicate and transfected for 48 h and then washed with PBS. Cell apoptosis was assessed using an Annexin-V-FITC/PI Apoptosis Detection Kit (Invitrogen, Thermo Fisher Scientific, USA) according to the manufacturer’s instructions. Early apoptotic cells were identified as PI negative and FITC Annexin V positive, and cells that were in late apoptosis were both FITC Annexin V and PI positive. The stained cells were examined with a flow cytometer (BD Biosciences, USA), and apoptosis was analyzed by using ModFit software.

### Western blot analysis

Human GC tissues or cultured cells were lysed using RIPA lysis buffer (Invitrogen, Carlsbad, CA, USA). Total protein was separated using 10% SDS polyacrylamide gels and transferred to nitrocellulose membranes (Roche Diagnostics, Basel, Switzerland). The membranes were incubated at 4 °C with primary antibodies that included mouse monoclonal anti-RASSF9 (1:1000; Cell Signaling Technology, USA), mouse monoclonal anti-p-AKT (1:1000; Cell Signaling Technology, USA), rabbit monoclonal anti-AKT (1:1000; Cell Signaling Technology, USA), rabbit monoclonal anti-Cyclin D1 (1:1000; Cell Signaling Technology, USA), rabbit monoclonal anti-CDK2 (1:1000; Cell Signaling Technology, USA), rabbit monoclonal anti-Bcl-2 (1:2000; Cell Signaling Technology, USA), rabbit monoclonal anti-Bax (1:2000; Cell Signaling Technology, USA), and mouse monoclonal anti-GAPDH (1:5000; Santa Cruz Biotechnology, CA, USA). The membranes were subsequently treated with ECL reagent (Pierce, USA) for chemiluminescence detection. The luminescent signal was measured by a CCD camera and recorded and quantified with Syngene GBox (Syngene, UK).

### Statistical analysis

The experiments were performed minimally in triplicate. All data were analyzed using SPSS 22.0 software (SPSS, Inc., Chicago, IL, USA). The data are presented as the mean ± SEM from at least 3 experiments. The statistical significance of differences between groups was analyzed with one-way ANOVA or Wilcoxon test. Correlation analysis between miR-1269 and RASSF9 in human GC tissues was performed using Spearman’s correlation analysis. Values of p < 0.05 were considered to indicate statistically significant differences.

## Results

### miR-1269 is significantly upregulated in human GC tissues and cell lines

**Fig. 1:**
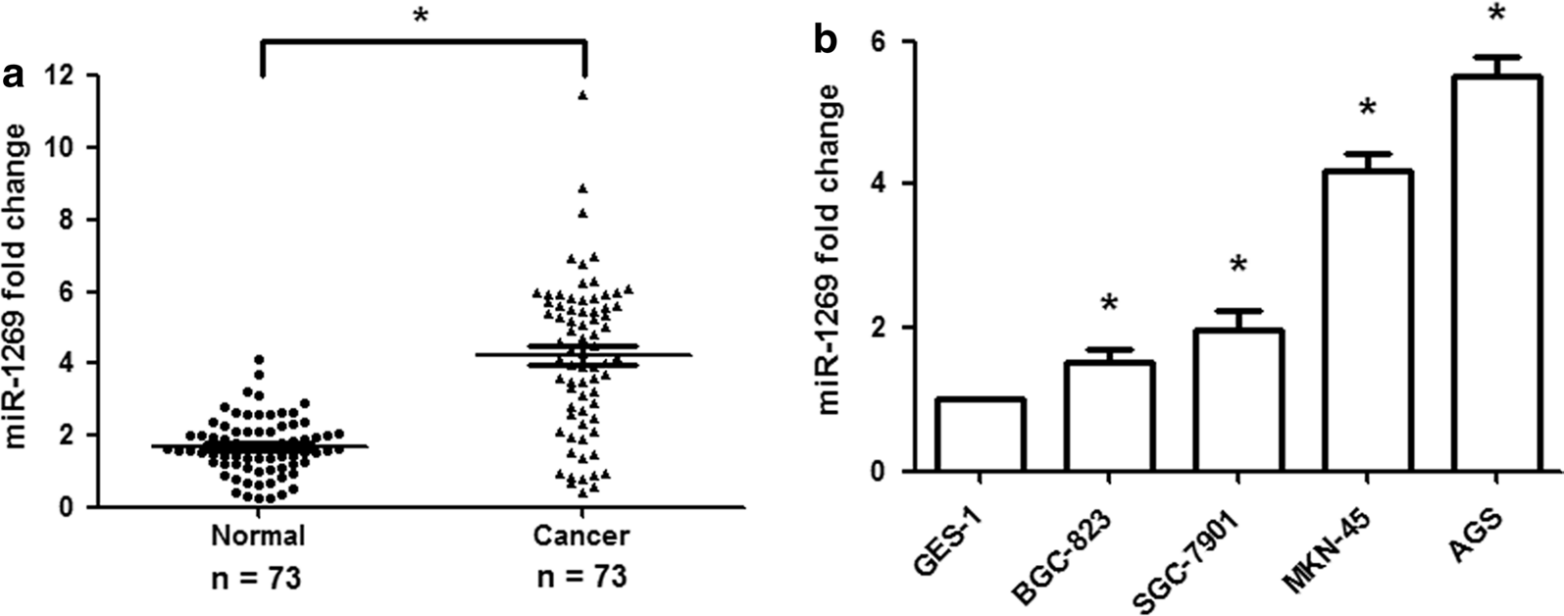
miR-1269 is upregulated in human GC tissues and cell lines. **a** qRT-PCR showed that miR-1269 expression was significantly increased in GC tissues compared with normal gastric tissues. **b** miR-1269 expression was significantly increased in GC cell lines (BGC-823, SGC-7901, MKN-45 and AGS) compared with a normal gastric epithelial cell line (GES-1). Wilcoxon test, *p < 0.01

qRT-PCR was performed to detect the expression of miR-1269 in 73 primary GC tissues, 73 matched adjacent normal tissues, and human GC cell lines (BGC-823, SGC-7901, MKN-45 and AGS). The results revealed that miR-1269 expression was remarkably upregulated in 80.8% (59/73) of the GC tissues compared to the normal tissues (Fig. 1a; p < 0.01). Furthermore, miR-1269 expression was significantly upregulated in human GC cell lines compared with normal gastric epithelial cells (GES-1) (Fig. 1b; p < 0.01). These findings indicated that miR-1269 acts as an oncogene in GC and has potential as an effective biomarker.

### miR-1269 facilitates GC cell growth, induces cell cycle transition and inhibits apoptosis

**Fig. 2:**
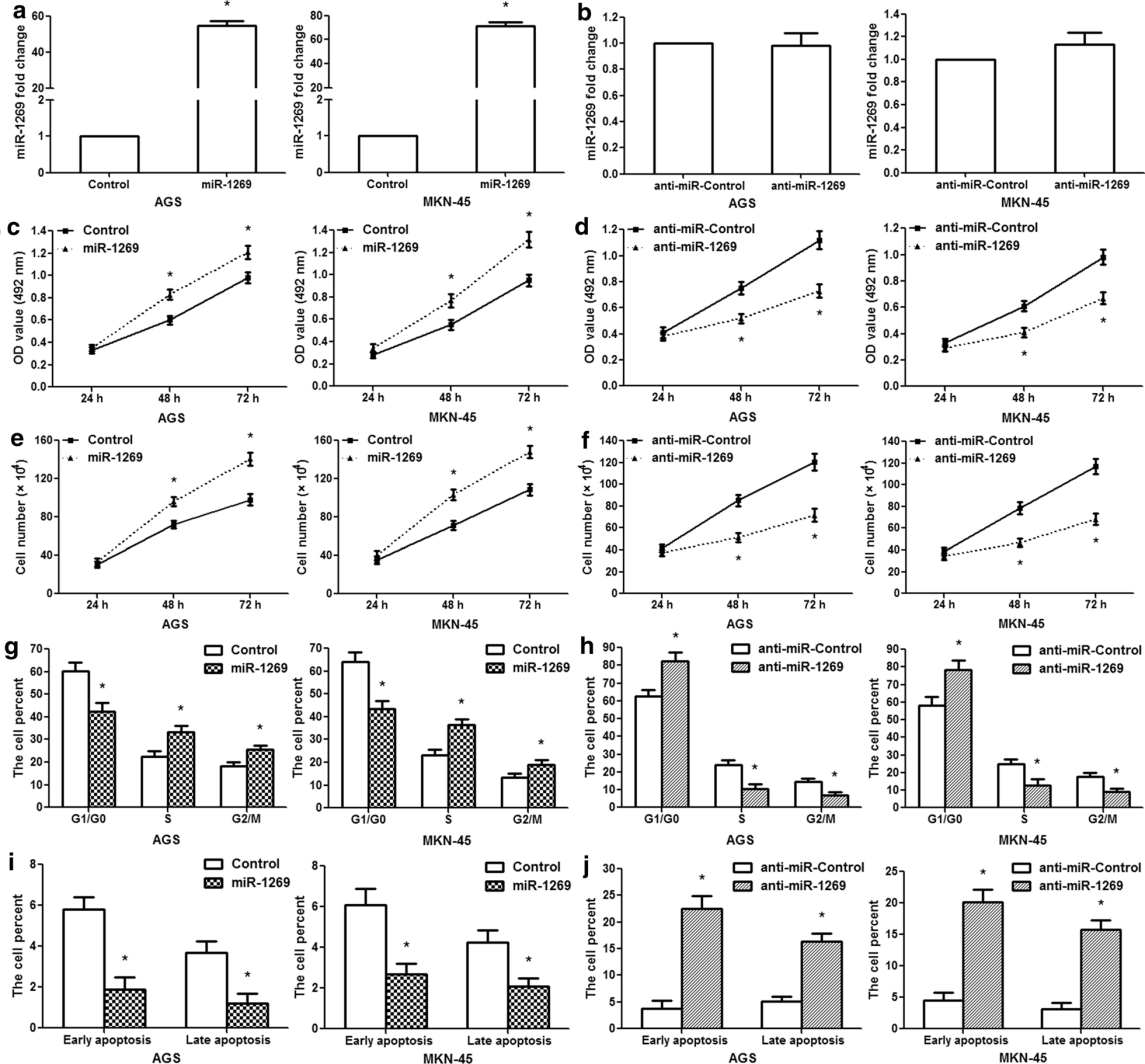
miR-1269 promotes human GC AGS/MKN-45 cell proliferation and inhibits apoptosis. **a** miR-1269 expression was examined in AGS/MKN-45 cells after miR-1269 overexpression. **b** miR-1269 expression was detected in AGS/MKN-45 cells after anti-miR-1269 treatment. **c** MTT assay revealed that miR-1269 overexpression promoted cell activity at 48 and 72 h after transfection. **d** MTT assay showed that anti-miR-1269 suppressed cell activity at 48 and 72 h after transfection. **e** Cell counting assay showed that miR-1269 overexpression increased GC cell proliferation. **f** Cell counting assays revealed that anti-miR-1269 suppressed GC cell growth. **g** Flow cytometry analysis of the cell cycle. The histogram represents the proportion of cells in the G0/G1, S and G2/M phases after miR-1269 overexpression. **h** The ratio of cells in the G0/G1, S and G2/M phases after anti-miR-1269 transfection. **i** Flow cytometry analysis of cell apoptosis. The data showed the ratios of early and late apoptosis after miR-1269 overexpression. **j** The data revealed the proportions of cells in early and late apoptosis after anti-miR-1269 transfection. Wilcoxon test, *p < 0.01, n = 3

To explore the role of miR-1269 in human GC, AGS/MKN-45 cells were transfected with the miR-1269 precursor expression vector, a control empty vector, miR-1269 antisense oligonucleotides, or the negative control. qRT-PCR was conducted to detect the expression of miR-1269 after treatment. miR-1269 expression was markedly increased in cells transfected with the miR-1269 vector compared to cells transfected with the control vector (p < 0.01); however, there were no significant differences between the anti-miR-1269 group and the anti-miR-Control group (Fig. 2a, b). The MTT assay showed that miR-1269 overexpression significantly promoted the proliferation of AGS/MKN-45 cells at 48 and 72 h after treatment (Fig. 2c; p < 0.01), while anti-miR-1269 suppressed AGS/MKN-45 cell growth at 48 and 72 h after treatment (Fig. 2d; p < 0.01). There was a similar trend in the cell counting assay. miR-1269 overexpression increased AGS/MKN-45 cell proliferation, while anti-miR-1269 inhibited cell proliferation (Fig. 2e, f; p < 0.01). Since the cell cycle is involved in the regulation of cell growth, we measured this process using a flow cytometer. The finding revealed that miR-1269 overexpression significantly decreased the G1/G0 phase population and increased the S and G2/M phase population in AGS/MKN-45 cells (Fig. 2g; p < 0.01); however, inhibition of miR-1269 resulted in a marked accumulation of cells in the G1/G0 phase and a reduction of cells in the S and G2/M phase (Fig. 2h; p < 0.01). Apoptosis analysis revealed that the proportion of cells in early- and late-stage apoptosis remarkably decreased in the miR-1269 overexpression group but significantly increased in the anti-miR-1269 group (Fig. 2i, j; p < 0.01). Our findings demonstrated that miR-1269 promoted GC cell proliferation, induced G1-S cell cycle transition and suppressed apoptosis.

### RASSF9 is a target gene of miR-1269

**Fig. 3:**
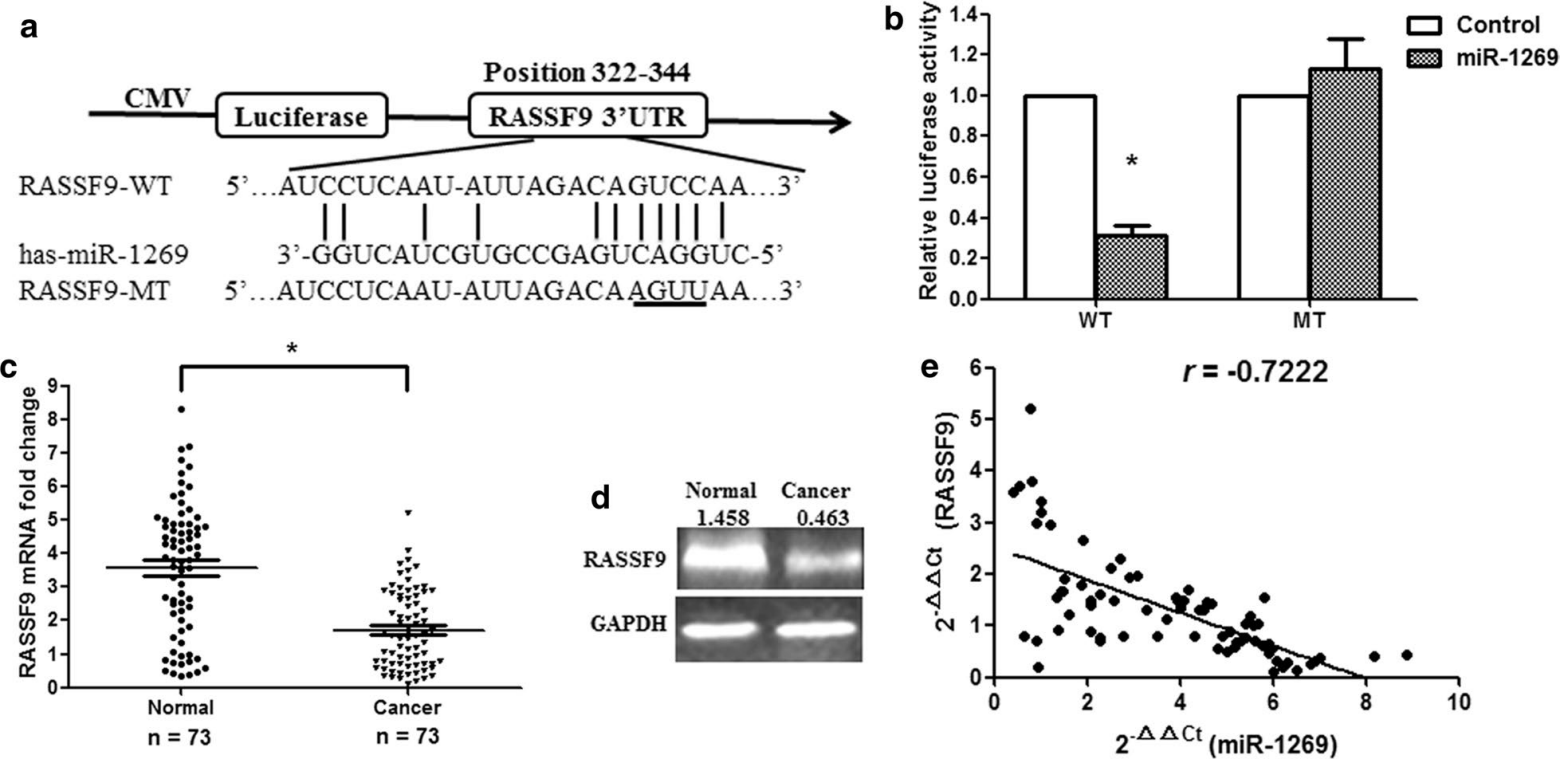
miR-1269 directly targets the RASSF9 gene. **a** Bioinformatics analysis predicted interactions between miR-1269 and its binding sites in the 3′-UTR of RASSF9. **b** Luciferase activity was measured by the dual-luciferase assay. **c** RASSF9 mRNA expression in human GC tissues. **d** RASSF9 protein levels were examined by Western blotting (n = 10). **e** miR-1269 and RASSF9 levels were negatively correlated. The 2^−∆∆Ct^ values of miR-1269 and negatively were subjected to a Spearman correlation analysis (n = 73, r = − 0.7222, p < 0.0001). Wilcoxon test, *p < 0.01

TargetScanHuman 7.2 and miRBase were used to predict a large number of possible target genes of miR-1269. Dual-luciferase assays showed that, among the candidate genes, RASSF9 was the most affected target gene of miR-1269; therefore, RASSF9 was selected for further study. We found that there was a binding site for miR-1269 in the 3′-UTR of the RASSF9 mRNA located from 322 to 344 bp (Fig. 3a). To determine whether miR-1269 directly targets RASSF9, a dual-luciferase reporter assay was performed with the WT and MT 3′-UTR of RASSF9. HEK293T cells were cotransfected with reporter plasmids and pre-miR-1269 or the pmirGLO empty vector (control). Pre-miR-1269/WT-RASSF9-UTR-transfected cells showed a remarkable reduction in luciferase activity (p < 0.01), while pre-miR-1269 failed to inhibit the relative luciferase activity MT-RASSF9-UTR-transfected cells (Fig. 3b), suggesting that miR-1269 directly targets the 3′-UTR of RASSF9. Next, we measured RASSF9 expression at the mRNA and protein levels. The results showed that the expression of RASSF9 was markedly downregulated at both the mRNA and protein levels in GC tissues compared to that in adjacent normal tissues (Fig. 3c, d; p< 0.01). The effect of miR-1269 on RASSF9 was evaluated based on the data obtained from qRT-PCR. A significant negative correlation was identified between RASSF9 mRNA and miR-1269 expression (Fig. 3e; n = 73, r = − 0.7222, p < 0.001, Spearman’s correlation).

### miR-1269 promotes GC cell proliferation and suppresses apoptosis through the AKT and Bcl-2/Bax signaling pathways by targeting RASSF9

**Fig. 4:**
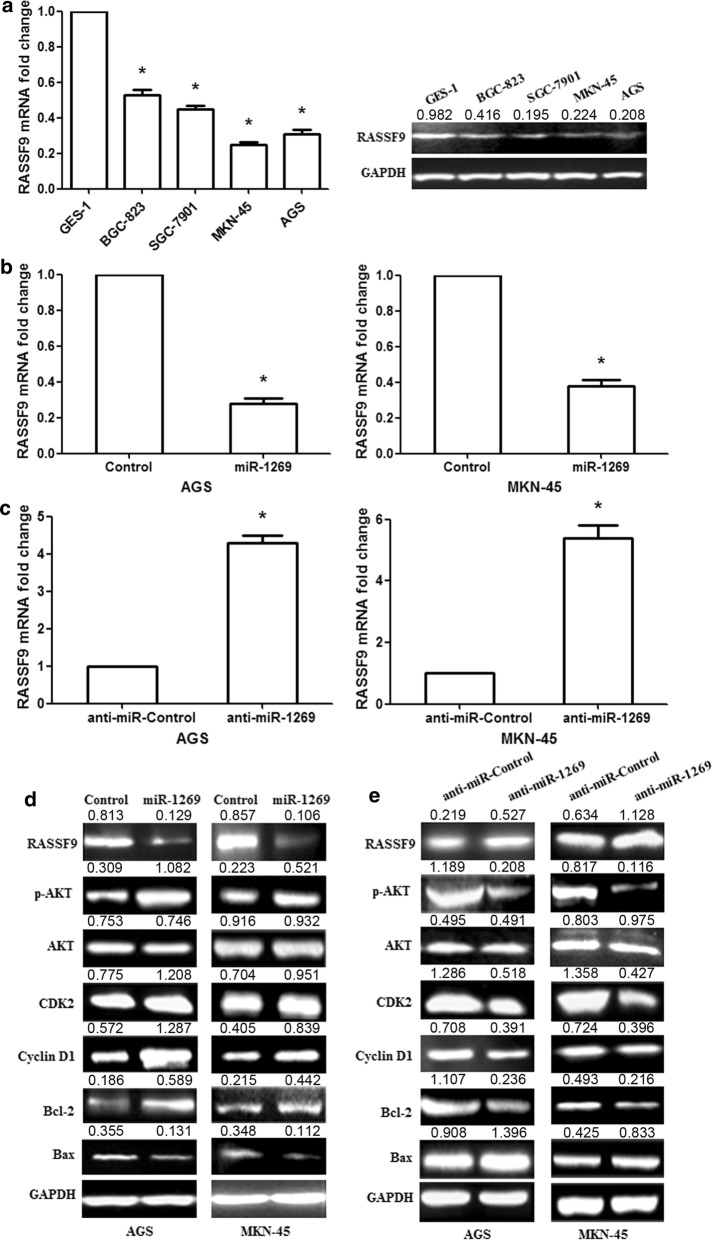
miR-1269 regulates the AKT and Bcl-2/Bax signaling pathways in human GC cells by targeting RASSF9. **a** RASSF9 mRNA and protein expression levels were significantly decreased in GC cell lines (BGC-823, SGC-7901, MKN-45 and AGS) compared with a normal gastric epithelial cell line (GES-1). **b** RASSF9 mRNA was examined in AGS/MKN-45 cells after miR-1269 overexpression. **c** RASSF9 mRNA was determined in AGS/MKN-45 cells after anti-miR-1269 treatment. **d** miR-1269 overexpression promoted the expression of the p-AKT, Cyclin D1, CDK2 and Bcl-2 proteins and inhibited RASSF9 and Bax expression in AGS/MKN-45 cells. **e** Anti-miR-1269 inhibited p-AKT, Cyclin D1, CDK2 and Bcl-2 protein expression and increased RASSF9 and Bax expression. Wilcoxon test, *p < 0.01

RASSF9 mRNA and protein expression were significantly downregulated in human GC cell lines compared with normal gastric epithelial cells (GES-1) (Fig. 4a; p< 0.01). miR-1269 overexpression significantly downregulated the mRNA expression of RASSF9 in AGS/MKN-45 cells, while anti-miR-1269 increased RASSF9 mRNA expression (Fig. 4b, c; p< 0.01). Similar results were also observed at the protein level (Fig. 4d, e). To further research the possible mechanisms of miR-1269-mediated regulation of cell growth, cell cycle progression and apoptosis, we examined related protein expression in the Bcl-2/Bax and AKT signaling pathways. The results showed that miR-1269 overexpression increased p-AKT, Cyclin D1, CDK2 and Bcl-2 protein expression and decreased Bax protein levels in AGS/MKN-45 cells (Fig. 4d). In contrast, anti-miR-1269 inhibited p-AKT, Cyclin D1, CDK2 and Bcl-2 protein expression and promoted Bax expression (Fig. 4e). These results indicated that miR-1269 could modulate GC cell proliferation and apoptosis via the regulation of the AKT and Bcl-2/Bax signaling pathways.

### Silencing of RASSF9 facilitates GC cell proliferation and inhibits apoptosis

**Fig. 5:**
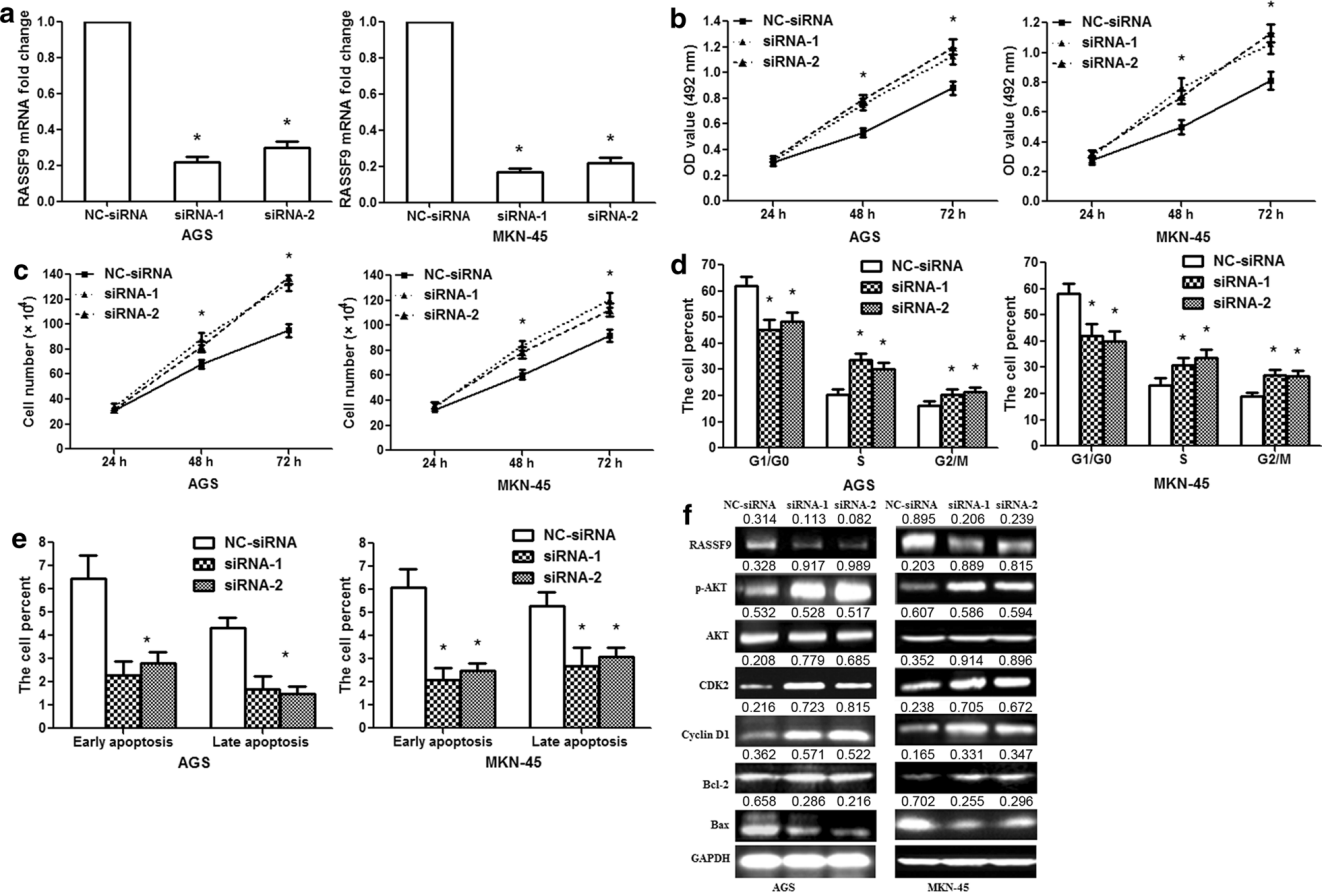
RASSF9 siRNAs promote the proliferation of human GC cells. **a** The results showed the knockdown efficiency of RASSF9 siRNA-1/2 in AGS/MKN-45 cells at 24 h after transfection. **b** MTT assay showed that RASSF9 siRNA-1/2 increased the activity of AGS/MKN-45 cells at 48 and 72 h. **c** Cell counting assay revealed that RASSF9 siRNA-1/2 promoted AGS/MKN-45 cell proliferation at 48 and 72 h. **d** Flow cytometric analysis showed the percentage of AGS/MKN-45 cells in the G0/G1, S and G2/M phases. The proportion of G0/G1 phase cells decreased after RASSF9 siRNA-1/2 transfection, and the proportion of S and G2/M phase cells increased. **e** The data showed the percentage of early and late apoptosis after RASSF9 siRNA-1/2 transfection. **f** RASSF9, p-AKT, Cyclin D1, CDK2, Bcl-2 and Bax protein expression levels were measured 48 h after RASSF9 siRNA-1/2 transfection. Wilcoxon test, *p < 0.01, n = 3

Since miR-1269 regulated cell proliferation and apoptosis in human GC cells and RASSF9 was validated as a direct target of miR-1269; in the next experiments, RASSF9 was knocked down in AGS/MKN-45 cells by RNA interference to validate its involvement in the pro-tumor functions of miR-1269. RASSF9 mRNA expression was specifically knocked down in AGS/MKN-45 cells using siRNA-1/2 (Fig. 5a; p< 0.01). Silencing RASSF9 increased cell activity at 48 and 72 h after transfection (Fig. 5b; p< 0.01). The cell counting assay showed that silencing RASSF9 increased AGS/MKN-45 cell proliferation (Fig. 5c; p< 0.01). Silencing RASSF9 decreased the G1/G0 phase population and increased the S and G2/M phase populations among AGS/MKN-45 cells (Fig. 5d; p< 0.01). In addition, silencing RASSF9 suppressed apoptosis in AGS/MKN-45 cells (Fig. 5e; p< 0.01). Next, we analyzed the knockdown efficiency of RASSF9 siRNA-1/2. RASSF9 protein expression was significantly downregulated in the siRNA group; RASSF9 siRNA-1/2 increased p-AKT, Cyclin D1, CDK2 and Bcl-2 protein expression and decreased Bax protein levels in AGS/MKN-45 cells (Fig. 5f). The results were similar to those obtained after miR-1269 overexpression, suggesting similar effects of RASSF9 knockdown and miR-1269 overexpression.

### RASSF9 overexpression suppresses GC cell growth and induces apoptosis

**Fig. 6:**
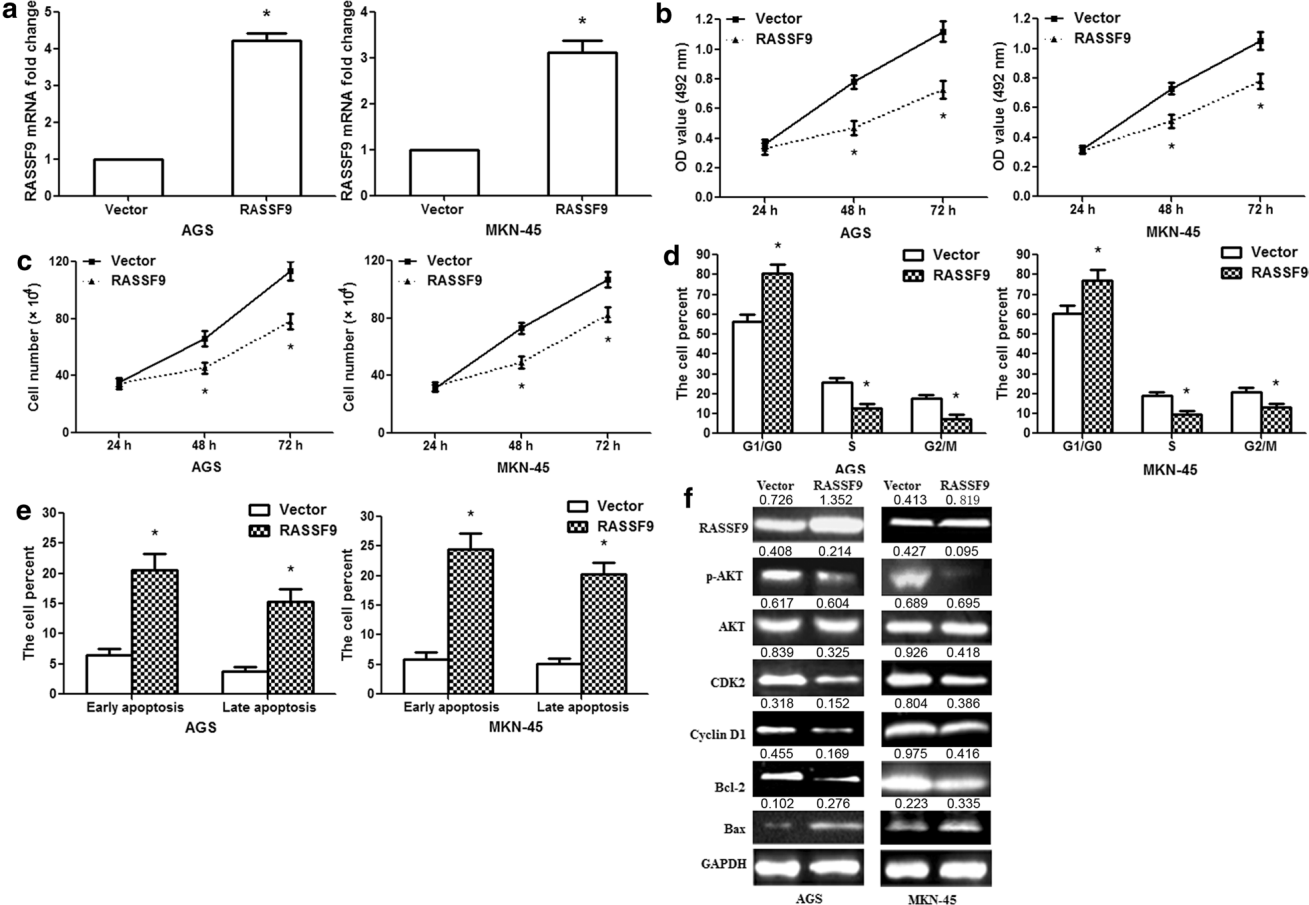
RASSF9 overexpression inhibits the proliferation of human GC cells. **a** RASSF9 overexpression promoted RASSF9 mRNA levels in AGS/MKN-45 cells. **b** MTT assay showed that RASSF9 overexpression decreased AGS/MKN-45 cell activity at 48 and 72 h. **c** Cell counting assay showed that RASSF9 overexpression inhibited AGS/MKN-45 cell growth. **d** The cell cycle was measured in AGS/MKN-45 cells at 48 h. **e** Apoptosis was examined in AGS/MKN-451 cells at 48 h. **f** RASSF9, p-AKT, AKT, Cyclin D1, CDK2, Bcl-2 and Bax expression levels were detected after transfection with the RASSF9 overexpression vector. Wilcoxon test, *p < 0.01, n = 3

To further determine that miR-1269 performed an oncogenic function via RASSF9, we constructed a RASSF9 overexpression vector. In AGS/MKN-45 cells, RASSF9 overexpression efficiently increased RASSF9 mRNA levels (Fig. 6a; p< 0.01). Based on the results of cell viability and the cell counting assay, RASSF9 overexpression inhibited AGS/MKN-45 cell proliferation (Fig. 6b, c; p< 0.01). RASSF9 overexpression increased the proportion of cells in G1 phase and reduced the proportion of cells in S and G2/M phases (Fig. 6d; p< 0.01). RASSF9 overexpression dramatically promoted early and late apoptosis (Fig. 6e; p< 0.01). We further analyzed the RASSF9 overexpression efficiency. RASSF9 protein expression was clearly upregulated in the RASSF9 overexpression vector group, and RASSF9 overexpression inhibited p-AKT, Cyclin D1, CDK2 and Bcl-2 protein expression and promoted Bax protein expression in AGS/MKN-45 cells (Fig. 6f). Therefore, miR-1269 could regulate human GC cell progression by targeting RASSF9 via the AKT and Bcl-2/Bax signaling pathways.

### Overexpression of RASSF9 reversed the effects of miR-1269 on GC cells

**Fig. 7:**
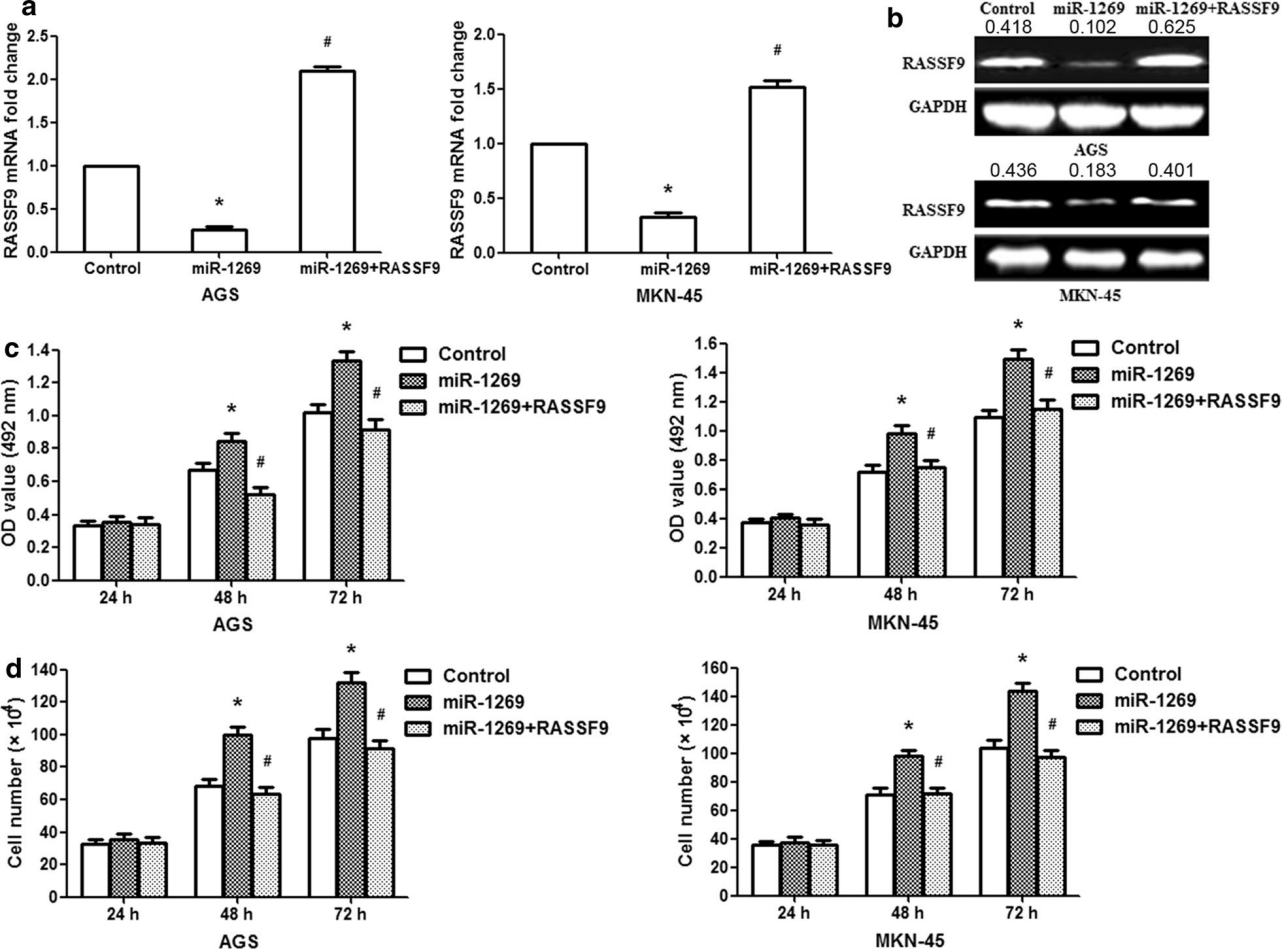
RASSF9 overexpression rescues the miR-1269-induced cellular phenotypes in GC cells. **a** RASSF9 overexpression rescued RASSF9 mRNA expression levels reduced by miR-1269. **b** RASSF9 overexpression rescued RASSF9 protein expression levels reduced by miR-1269. **c** MTT assay was performed to examine the growth of AGS/MKN-45 cells after cotransfection with RASSF9 and miR-1269 expression vectors. **d** Cell counting assay was used to measure AGS/MKN-45 cell proliferation following cotransfection with RASSF9 and miR-1269. One-way ANOVA, *p < 0.01, compared with the control group; #p < 0.01, compared with the miR-1269 overexpression group, n = 3

Next, the RASSF9 overexpression vector was cotransfected with miR-1269 into AGS/MKN-45 cells. Overexpression of RASSF9 in AGS/MKN-45 cells rescued the RASSF9 expression levels reduced by miR-1269 (Fig. 7a, b; p< 0.01). After cotransfection of the miR-1269 and RASSF9 vectors, we found that the overexpression of RASSF9 counterbalanced the oncogenic effect of miR-1269 on the proliferation of GC cells (Fig. 7c, d; p< 0.01). These results further demonstrated that miR-1269 played an oncogenic role by targeting RASSF9.

## Discussion

In recent years, a wide variety of studies have demonstrated that miRNAs play crucial roles in regulating the biological characteristics of GC, such as cell proliferation, apoptosis, invasion, migration, metabolism, and metastasis [23–26]. Due to their important roles in GC, miRNAs have been proposed as prospective biomarkers and therapeutic targets of GC [27]. Although the clinical significance of miRNAs in GC has been well recognized, the roles and molecular mechanisms of dysregulated miRNAs remain largely unknown. Hence, validating miRNAs and elucidating their precise biological functions and characteristics in GC will help in the search for new targets in GC diagnosis and therapy. Recent studies have found that miR-1269 expression is upregulated and promotes tumor cell survival and proliferation in lung cancer and hepatocellular carcinoma [17, 28]. In the present study, we found that the expression of miR-1269 was upregulated in both GC tissues and cell lines. miR-1269 overexpression promoted GC cell proliferation by inducing the G1-S phase transition and inhibited cell apoptosis in vitro. Inhibition of miR-1269 suppressed GC cell growth and induced cell apoptosis. Overall, miR-1269 acts as an oncogene in GC and has the potential to be a new diagnostic marker and therapeutic target for this disease.

In this study, RASSF9 was identified as a direct target of miR-1269. Among RASSF family members, RASSF7 functions as an oncogene in lung cancer progression by inhibiting the phosphorylation of mammalian Ste20-like kinase 1, large tumor suppressor kinase 1 and yes-associated protein [29]. RASSF8 and RASSF10 suppress cell proliferation, migration and invasion in gastric cancer, lung cancer, liver cancer, cervical cancer, colorectal cancer and esophageal squamous cell carcinoma [30–34]. Recently, it was reported that RASSF9 suppresses cell proliferation in breast cancer [22]. However, its function in other cancers remains unclear. The present study identified the downregulation of RASSF9 in GC tissues and an inverse correlation between RASSF9 expression and miR-1269 expression. miR-1269 overexpression inhibited RASSF9 3′-UTR luciferase reporter activity, which could be abrogated by mutation of the miR-1269 binding site. The overexpression of miR-1269 also inhibited RASSF9 expression at both the mRNA and protein levels in GC cells. Silencing RASSF9 promoted GC cell proliferation, induced G1-S phase transition, and inhibited apoptosis. Overexpression of RASSF9 inhibited cell proliferation and G1-S phase transition and induced apoptosis. In brief, miR-1269 acts as a positive regulator or an oncogene for cell proliferation, which is partially mediated by suppressing RASSF9 expression.

The PI3K/AKT pathway is one of the most potent proliferation pathways in cancer [35]. Dysregulation of the PI3K/AKT pathway is involved in tumorigenesis and progression, especially in lung, esophagus, gastric, renal, colorectal, liver and breast cancer [36, 37]. It has been found that AKT activity is correlated with various clinicopathological features of cancers [38]. AKT regulates the roles of certain substrates related to the cell cycle by directly phosphorylating target proteins or indirectly controlling protein expression [36]. CDK2 and Cyclin D1, the downstream genes of AKT, are important transcriptional factors in the G0/G1 phase [39]. Cyclin A-CDK2 and Cyclin D-CDK4/6 protein kinase complexes are crucial cell cycle regulators and control cell cycle transition from the G1/G0 phase to the S phase [40]. D-Cyclins drive entry into the S phase by releasing E2F transcription factors after extracellular mitogenic stimulation. It has been reported that Cyclin A-CDK2 protein kinase complexes regulate cell growth and the cell cycle in many human cancers [41, 42]. The present study demonstrated that miR-1269 overexpression and RASSF9 siRNA upregulated CDK2 and Cyclin D1 expression and induced the G1-S phase transition by activating the AKT signaling pathway, while anti-miR-1269 and RASSF9 overexpression downregulated the expression of CDK2 and Cyclin D1 and resulted in G1-S phase transition arrest by inhibiting the PI3 K/AKT signaling pathway. These findings indicate that miR-1269 may upregulate the expression of CDK2 and Cyclin D1 and facilitate G1-S phase transition through activation of the AKT signaling pathway by targeting RASSF9.

The growth rate of human cancers is involved in cell proliferation and cell death. An imbalance between apoptosis-related proteins, such as Bcl-2 and Bax, can induce dysregulation of apoptosis, which can lead to onco-genesis and cancer progression. Bax, a proapoptotic factor, may cause the release of cytochrome c resulting in mitochondrial dysfunction. In contrast, Bcl-2, an antiapoptotic factor, protects the outer membrane and preserves its integrity by suppressing the release of cytochrome [43]. Thus, a criti-cal determinant of the intrinsic apoptotic pathway is the balance between Bax and Bcl-2 expression [44]. Our findings revealed that miR-1269 overexpression and RASSF9 knockdown suppressed GC cell apoptosis by regulating the Bax/Bcl-2 signaling pathway, and anti-miR-1269 and RASSF9 overexpression induced apoptosis by controlling the Bax/Bcl-2 signaling pathway. These findings suggest that miR-1269 may inhibit GC cancer cell apoptosis by regulating the Bax/Bcl-2 signaling pathway via RASSF9.

## Conclusions

In summary, our study demonstrated that miR-1269 acts as an oncogene in GC. We discovered that miR-1269 is significantly upregulated in human GC tissues and cell lines. miR-1269 facilitated GC cell proliferation by activating the PI3K/AKT signaling pathway and inhibited cell apoptosis through the regulation of the Bax/Bcl-2 signaling pathway via RASSF9. These findings suggest that miR-1269 plays a key role in GC progression and may represent a potential new target for GC therapy.

## Data Availability

The datasets used in this study are available from the corresponding author upon reasonable request.

## Abbreviations

GC: gastric cancer
miRNAs: microRNAs
miR-1269: microRNA-1269
MT: mutant type
CDK2: cyclin-dependent kinase 2
qRT-PCR: quantitative real-time PCR
RASSF9: Ras-association domain family 9
RPMI-1640: Roswell Park Memorial Institute-1640
siRNA: small interfering RNA
WT: wild type
3′-UTR: 3′-untranslated region
